# Assessment of antibiotic resistance and biofilm formation of *Enterococcus* species isolated from different pig farm environments in Poland

**DOI:** 10.1186/s12866-023-02834-9

**Published:** 2023-03-30

**Authors:** Katarzyna Grudlewska-Buda, Krzysztof Skowron, Justyna Bauza-Kaszewska, Anna Budzyńska, Natalia Wiktorczyk-Kapischke, Monika Wilk, Magdalena Wujak, Zbigniew Paluszak

**Affiliations:** 1 Department of Microbiology, Ludwik Rydygier Collegium Medicum in Bydgoszcz, Nicolaus Copernicus University in Toruń, Toruń, Poland; 2 Department of Microbiology and Food Technology, Bydgoszcz University of Science and Technology, Bydgoszcz, Poland; 3 Department of Medicinal Chemistry, Ludwik Rydygier Collegium Medicum in Bydgoszcz, Nicolaus Copernicus University in Toruń, Toruń, Poland

**Keywords:** *Enterococcus* spp., Antibiotic resistance, VRE, MIC values, Biofilm formation, Pig farms

## Abstract

**Background:**

*Enteroccocus* spp. are human opportunistic pathogens causing a variety of serious and life-threating infections in humans, including urinary tract infection, endocarditis, skin infection and bacteraemia. Farm animals and direct contact with them are important sources of *Enterococcus faecalis* (EFA) and *Enterococcus faecium* (EFM) infections among farmers, veterinarians and individuals working in breeding farms and abattoirs. The spread of antibiotic-resistant strains is one of the most serious public health concerns, as clinicians will be left without therapeutic options for the management of enterococcal infections. The aim of the study was to evaluate the occurrence and antimicrobial susceptibility of EFA and EFM strains isolated from a pig farm environment and to determine the biofilm formation ability of identified *Enterococcus* spp. strains.

**Results:**

A total numer of 160 enterococcal isolates were obtained from 475 samples collected in total (33.7%). Among them, 110 of genetically different strains were identified and classified into EFA (82; 74.5%) and EFM (28; 25.5%). Genetic similarity analysis revealed the presence of 7 and 1 clusters among the EFA and EFM strains, respectively. The highest percentage of EFA strains (16; 19.5%) was resistant to high concentrations of gentamicin. Among the EFM strains, the most frequent strains were resistant to ampicillin and high concentrations of gentamicin (5 each; 17.9%). Six (7.3%) EFA and 4 (14.3%) EFM strains showed vancomycin resistance (VRE - Vancomycin-Resistant *Enterococcus*). Linezolid resistance was found in 2 strains of each species. The multiplex PCR analysis was performed to identify the vancomycin resistant enterococci. *vanB, vanA and vanD* genotypes were detected in 4, 1 and 1 EFA strains, respectively. Four EFA VRE-strains in total, 2 with the *vanA* and 2 with the *vanB* genotypes, were identified. The biofilm analysis revealed that all vancomycin-resistant *E. faecalis* and *E. faecium* strains demonstrated a higher biofilm-forming capacity, as compared to the susceptible strains. The lowest cell count (5.31 log CFU / cm^2^) was reisolated from the biofilm produced by the vancomycin-sensitive strain EFM 2. The highest level of re-isolated cells was observed for VRE EFA 25 and VRE EFM 7 strains, for which the number was 7 log CFU / cm^2^ and 6.75 log CFU / cm^2^, respectively.

**Conclusions:**

The irrational use of antibiotics in agriculture and veterinary practice is considered to be one of the key reasons for the rapid spread of antibiotic resistance among microorganisms. Owing to the fact that piggery environment can be a reservoir of antimicrobial resistance and transmission route of antimicrobial resistance genes from commensal zoonotic bacteria to clinical strains, it is of a great importance to public health to monitor trends in this biological phenomenon.

## Introduction

*Enterococcus* spp. is a genus of Gram-positive, non-sporulating cocci, commonly found in natural environments (i.e. soil, water, plants), food products and in the digestive tract of humans and farmed animals [1–3]. Despite the fact that species comprising the gut microbiome are generally considered commensals, with an average number of 10^3^ to 10^7^ CFU in 1 g of faeces, some *Enterococcus* spp. are defined as potentially opportunistic pathogens causing life-threatening infections, which mainly affect hospitalized patients [4]. Transmission of enterococci to humans may also involve direct contact with animals and working in their environment. Therefore, individuals such as farmers, veterinarians and employees of breeding farms and slaughterhouses may be at higher risk of *Enterococcus* spp. infection and colonization. The species *E. faecalis* and *E. faecium*, most frequently isolated from humans, can cause urinary tract infections, skin infections (including burn and operative wounds) and infective endocarditis. These strains may be also responsible for sepsis and inflammation, e.g. in the cerebrospinal fluid [1, 5, 6]. In pigs, enterococci can cause serious infections, including sepsis preceded by high fever, rash and muscle flaccidity [6, 9]. In addition to *E. faecium* and *E. faecalis*, other species have been isolated from farm animals, including *E. hirae*, *E. durans*, *E. cecorum*, *E. casseliflavus*, *E. gallinarum* and *E. avium*, [2, 3, 6–8].

The presence of enterococci in the pig farm milieu has been repeatedly confirmed by isolating them from animal skin surface, faeces, feed and bedding as well as from various equipment for livestock handling [8, 10, 11]. These bacteria were also present in dust samples collected from the air in breeding pig facilities [12].

The risks associated with the presence of enterococci in the milieu of animal husbandry and livestock farming are not limited solely to their pathogenic potential. In comparison to other microorganisms of intestinal origin, enterococci demonstrate a relatively high resistance and adaptability to chemical and environmental stressors. As a consequence, they can adapt and survive under harsh conditions [2, 4, 13]. Enhanced resilience and survival in hostile environments is also attributed to their ability to form biofilms. This results in a reduced disinfection efficiency of surfaces colonized by the bacteria and consequently increased risk of potential infections [4, 14]. Nonetheless, expanding antibiotic resistance of enterococci is nowadays the greatest threat to human health worldwide.

Irrational use (overuse or misuse) of antibiotics in clinical medicine is the major factor contributing to the emergence of antibiotic-resistant bacterial strains in the environment. The routine application of antibiotics for growth promotion and bacterial infection prevention or control in livestock farming has also a great impact on this microbiological phenomenon. Administration of antibiotics in drinking water or feed for farm animals, despite the legitimacy of their use, results in the tissue-fluid penetration of these substances and consequently the deposition of their residues in meat and other raw materials of animal origin [11, 14–16]. Notably, there is evidence for a positive correlation between antibiotic administration and the occurence of antibiotic-resistant *Entreococcus* spp. strains [17].

*Enterococcus* spp. exhibit a high-level resistance to some classes of antibiotics, e.g. cephalosporins, and low susceptibility to penicillin. A low intrinsic sensitivity to penicillins is attributed particularly to *E. faecium* [16, 18, 19]. The rapid development of resistance mechanisms by enterococci is a result of a high mutation rate and a specific location of genes in mobile genetic elements (MGE) which are responsible for intracellular and intercellular DNA mobility [15, 16]. These features led to a gradual reduction in the sensitivity of these bacteria to other classes of antibiotics (β-lactams, aminoglycosides, tetracyclines, quinolones, macrolides, streptogramins and chloramphenicol) and the emergence of multi-drug resistant (MDR) strains. In response to the decreasing effectiveness of antibiotics used in established therapies, a natural and rational trend has emerged to test other antibiotics [3, 6, 16]. Satisfactory results in the treatment of infections caused by enterococci have been obtained for glycopeptides, in particular vancomycin. Unfortunately, starting from the 1980s, an increasing number of clinical enterococci strains with resistance to this antibiotic were reported. Currently, vancomycin-resistant enterococci (VRE) are considered to be one of the most important health-care associated (HA) multidrug-resistant pathogens. The rate of their occurrence in European countries and the United States shows an upward trend [5, 20]. Among the nine phenotypes of vancomycin-resistant enterococci strains (VanA, VanB, VanC, VanD, VanE, VanG, VanL, VanM, VanN), the VanA phenotype is dominant in most European countries and the United States [19]. The *vanA* gene cluster is often found in plasmids, which increases the risk of acquiring resistance to vancomycin and facilitates the dissemination of this trait among strains inhabiting a given environment [21].

The spread of VRE and MDR strains in the animal production environment may be due to the contact with animals carrying the bacteria and consumption or handling of contaminated food. However, transmission of antibiotic resistance from animals to humans poses a major global threat of growing concern about human and environment health. The process of horizontal transfer of drug resistance genes between bacteria can take place in any environment, including the gut of the host, and may involve both susceptible commensal enterococcal strains and pathogens such as *Listeria* spp. and *Staphylococcus aureus* [2, 15, 22].

Given the increase in the number of nosocomial infections caused by antibiotic-resistant enterococcal strains and the resulting serious health consequences, monitoring of their occurrence and antimicrobial resistance in the animal production environment is fully justified and essential to prevent the development of resistance. The aim of this study was to (a) assess the prevalence of *E. faecalis* and *E. faecium* in the environment of an industrial pig farm, (b) determine their drug susceptibility profiles, (c) to detect vancomycin resistant strains by phenotypic and genotypic methods and (d) the assessement of biofilom formation on stainless steel coupons using quantitative method.

## Results

From 475 samples collected, 121 (25.5%) were *E. faecalis*-positive and 39 (8.2%) were *E. faecium*-positive (Table 1). The greatest number of isolates, both *E. faecalis* (34; 28.1%) and *E. faecium* (11; 28.2%), were obtained from the weaned pigs sector. Irrespective of the sampling place, the highest number of positive samples was found in animal faeces (Table 1).

**Table 1:** Contribution of particular species of the *Enterococcus* spp. in the general population of isolates and strains obtained from different production sectors

Production sector	Species Sampling place	Number of samples	*E. faecalis*			*E. faecium*		
			isolates	strains	strain ID	isolates	strains	strain ID
Suckling pigs sector	Feeding passage	25	3	3	EFA 1–3	2	2	EFM 1–2
	Slurry channel	25	9	7	EFA 4–10	1	1	EFM 3
	Trough	25	2	1	EFA 11	1	1	EFM 4
	Faecal samples	25	13	10	EFA 12–21	6	4	EFM 5–8
Weaned pigs sector	Slurry channel	25	10	4	EFA 22–25	5	5	EFM 9–13
	Trough	25	8	1	EFA 26	-	-	-
	Faecal samples	25	16	12	EFA 27–38	6	3	EFM 14–16
Piglets sector	Feeding passage	25	2	2	EFA 39–40	-	-	-
	Trough	25	4	3	EFA 41–43	-	-	-
	Faecal samples	25	9	9	EFA 44–52	4	4	EFM 17–20
Porkers sector	Feeding passage	25	-	-	-	1	1	EFM 21
	Trough	25	6	4	EFA 53–56	-	-	-
	Faecal samples	25	11	11	EFA 57–67	2	1	EFM 22
Mating area	Feeding passage	25	3	1	EFA 68	-	-	-
	Slurry channel	25	8	6	EFA 69–74	3	1	EFM 23
	Trough	25	1	1	EFA 75	-	-	-
	Faecal samples	25	14	5	EFA 76–80	5	2	EFM 24–25
Loading ramp	Ramp	25	2	2	EFA 81–82	3	3	EFM 26–28
	Platform	25	-	-	-	-	-	-
	TOTAL	475	121	82		39	28	

## Analysis of genetic similarity

**Fig. 1:**
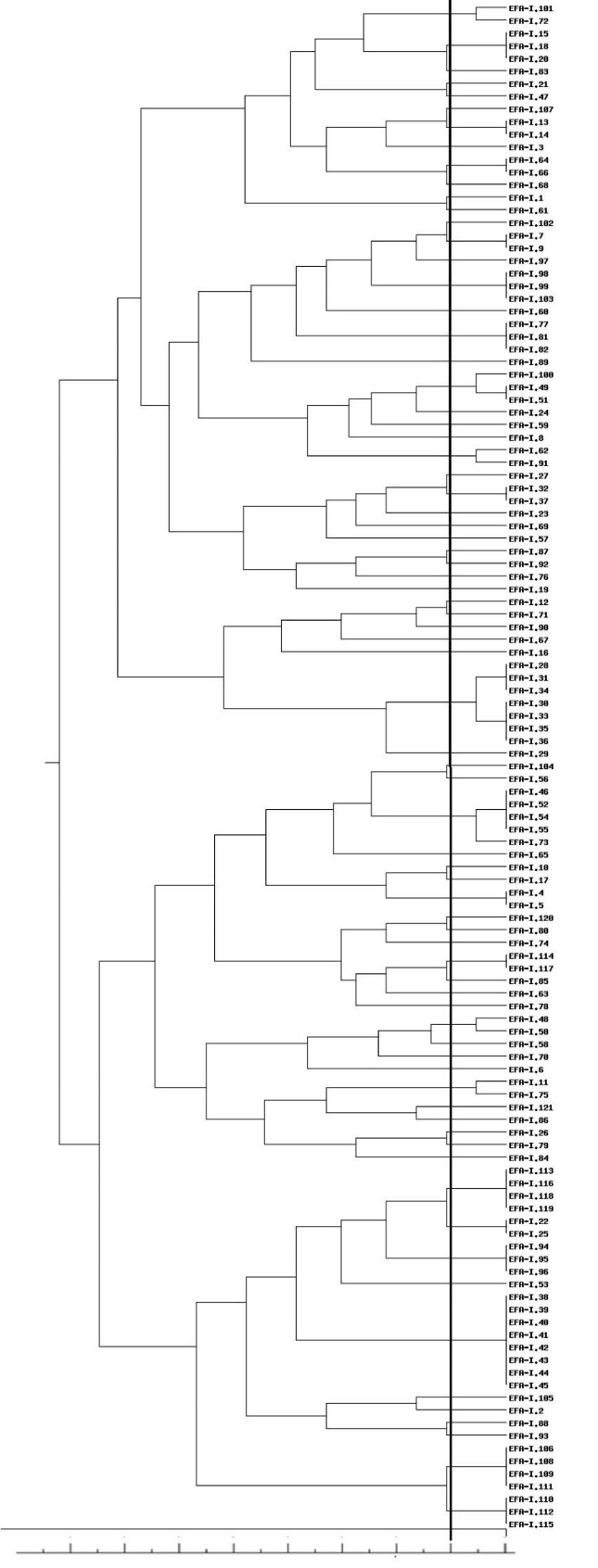
Dendrogram of genetic relatedness among 82 *E. faecalis* strains. The cut-off value to define the patterns was set at 80% similarity (marked as black line)

**Fig. 2:**
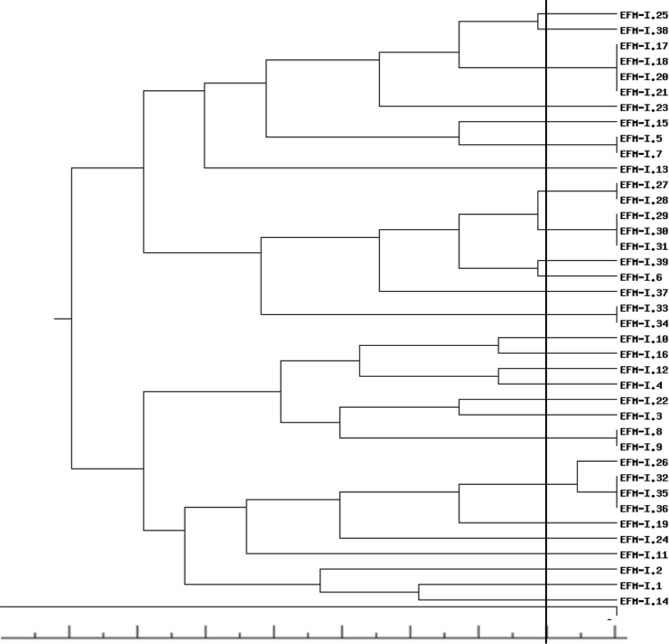
Dendrogram of genetic relatedness among 28 *E. faecium* strains. The cut-off value to define the patterns was set at 80% similarity (marked as black line)

Based on the analysis of genetic similarity, 82 and 28 genetically different strains of *E. faecalis* and *E. faecium*, respectively, were identified in the analyzed material (Figs. 1 and 2). Some isolates were genetically identical. For *E. faecalis* and *E. faecium*, 19 and 7 strains, respectively, were represented by more than one isolate (Figs. 1 and 2). All genetically identical isolates belonging to a given strain were obtained from samples taken from the same production sector. For the cut-off at a level of 80%, 1 cluster (C4 EFA) containing 7 strains, 1 cluster (C5 EFA) containing 5 strains, 1 cluster (C1 EFM) containing 4 strains, 1 cluster (C2 EFA) containing 3 strains and 4 clusters (C1 EFA, C3 EFA, C6 EFA, C7 EFA) containing 2 strains were found (Table 2).

**Table 2:** The cluster of genetic similarity determined for *E. faecalis* (n = 82) and *E. faecium* (n = 28) isolates at cut-off level of 80%

	RAPD cluster	Isolate number
*E. faecalis*	C1 EFA	EFA I.-101, EFA I-72
	C2 EFA	(EFA I.-49, EFA I.-51), EFA I.-100,
	C3 EFA	EFA I.-62, EFA I-91
	C4 EFA	(EFA I.-28, EFA I.-31, EFA I.-34), (EFA I.-30, EFA I-33, EFA I.-35, EFA I.-36)
	C5 EFA	(EFA I.-46, EFA I.-52, EFA I.-54, EFA I.-55), EFA I.-73
	C6 EFA	EFA I.-48, EFA I.-50
	C7 EFA	EFA I.-11, EFA I.-75
*E. faecium*	C1 EFM	EFM I.-26, (EFM I.-32, EFM I.-35, EFM I.-36)

Genetically identical isolates are presented in parentheses

## Analysis of antibiotic resistance

**Fig. 3:**
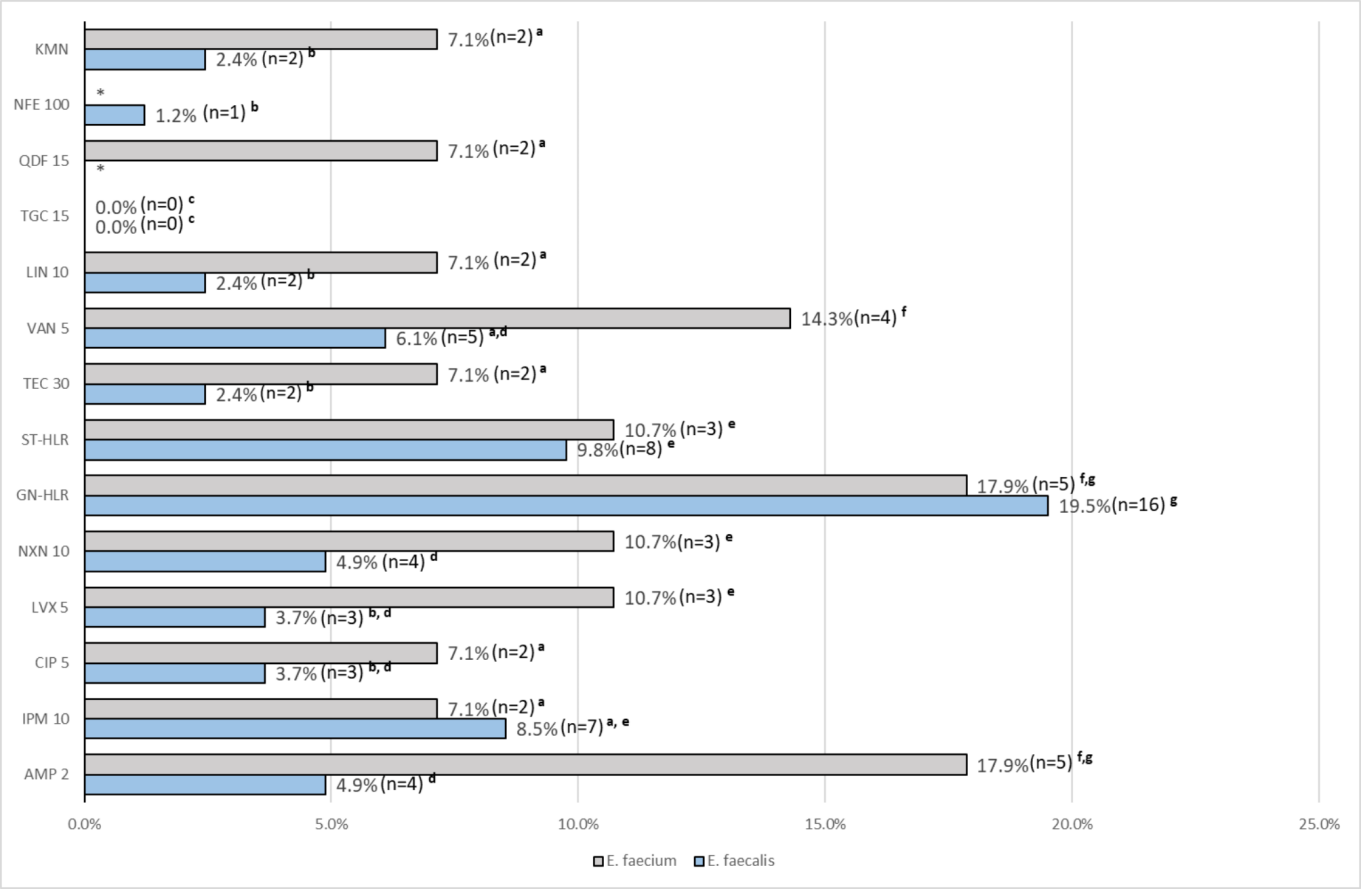
Proportion of antibiotic resistance-susceptibility profiles of *E. faecium* and *E. faecalis* strains isolated in the study (KMN—kanamycin, NFE—nitrofurantoin, QDF—quinupristin-dalfopristin, TGC—tigecycline, LIN—linezolid, VAN—vancomycin, TEC—teicoplanin, ST—streptomycin, GN—gentamycin, NXN—norfloxacin, LVX—levofoxacin, CIP—ciprofloxacin, IMP—imipenem, AMP—ampicillin), a,b,c,…—values marked with different letters differ statistically significant

The results from the analysis of the resistance of the isolated strains to antibiotics are presented in Fig. 3. In the studied population, all strains of both *E. faecalis* and *E. faecium* showed sensitivity to tigecycline. The highest percentage of *E. faecalis* strains (19.5%) was resistant to gentamicin (HLGR phenotype), which was a statistically significant difference as compared to other phenotypes, followed by strains resistant to streptomycin (9.8%) and imipenem (8.5%). Moreover, among strains sensitive to imipenem and ampicillin, 4.9% and 3.7% of isolates, respectively, were categorised as “susceptible, increased exposure” (formerly “intermediate”) to these antibiotics. In turn, *E. faecium* strains showed the highest resistance to gentamicin (HLGR phenotype, 17.9%), ampicillin (17.9%) and vancomycin (14.3%). The observed differences were statistically significant when compared to other drugs tested. Similar to *E. faecalis*, some *E. faecium* strains were categorised as “susceptible, increased exposure” to ampicillin and imipenem (7.1% each). High level of resistance to streptomycin (HLSR phenotype) was present in 9.8% and 10.7% of *E. faecalis* and *E. faecium* strains, respectively. In addition, 6.1% of *E. faecalis* strains and 14.3% of *E. faecium* strains showed vancomycin resistance. The number of teicoplanin insensitive strains was lower than VREs and was 2.4% and 7.1%, respectively. Moreover, we found 2 strains of each species resistant to linezolid. The differences in the frequency of resistance to linezolid, ampicillin, fluoroquinolones, glycopeptides and kanamycin between the EFM and EFA strains were statistically significant.

For all strains found to be vancomycin and teicoplanin resistant (according to determined MIC values), the presence of the *van* gene was confirmed by the multiplexPCR method. The *vanB* gene was detected in one *E. faecalis* strain which showed the sensitivity to vancomycin (MIC = 2 mg/L). The screening analysis for vancomycin resistance genes revealed that the *vanB* genotype was dominant in the *E. faecalis* population, occurring in 4 strains (4.9%). Moreover, one EFA strain (1.2%) showing both the *vanA* and *vanD* genotypes was isolated. In the case of *E. faecium*, two strains (7.1% each) demonstarted the *vanA* and *vanB* genotypes. In total, 26 drug susceptibility profiles were distinguished, of which 18 were found in *E. faecalis* and 11 in *E. faecium* (Table 3). The profile A, characterized by sensitivity to all tested antibiotics and chemotherapeutic agents, was dominant in both *E. faecalis* (67.1%) and *E. faecium* (53.6%) strains. Multidrug-sensitive strains and strains resistant to one of the tested antibiotics were the most prevalent in the *E. faecalis* isolates (17.1% of the population). As the number of drugs to which *E. faecalis* strains are insensitive increased, the percentage of these strains decreased. One strain of *E. faecalis* assigned with the ZY profile, showed resistance to the highest number (n = 7) of antibiotics. The analysis of *E. faecium* drug susceptibility profiles revelaed that 21.4% of strains were resistant to one antibiotic, 3.6% to two, and 7.1% of isolated strains each showed the resistance to three, four or five antibiotics. In the studied population, four (4.9%) *E. faecalis* and four (14.3%) *E. faecium* strains were identified as multidrug-resistant (MDR).

**Table 3:** Antibiotic resistance profiles of the investigated strains

Resistance profile	Antibiotic(s)	No. of *E. faecalis* (%)	No. of *E. faecium* (%)
A	Non-resistant to all antibiotics	55 (67.1)	15 (53.6)
B	R: AMP	0	2 (7.1)
C	R: IPM	2 (2.4)	0
D	R: VAN	1 (1.2)	1 (3.6)
E	R: GN-HLR	8 (25.0)	3 (10.7)
F	R: ST-HLR	2 (2.4)	0
G	R: LIN	1 (1.2)	0
H	R: NFE, GN-HLR	1 (1.2)	-
I	R: AMP, ST-HLR	1 (1.2)	0
J	R: VAN, GN-HLR	1 (1.2)	0
K	R: GN-HLR, ST-HLR	2 (2.4)	0
L	R: LVX, NXN	1 (1.2)	0
M	R: QDF, LIN	-	1 (3,6)
N	R: IPM, ST-HLR, KNM	1 (1.2)	0
O	R: AMP, IPM, LIN	1 (1.2)	0
P	R: VAN, GN-HLR, ST-HLR	1 (1.2)	0
R	R: CIP, LVX, NXN	0	1 (3.6)
S	R: LVX, NXN, QDF	-	1 (3.6)
T	R: AMP, GN-HLR, ST-HLR, KNM	0	1 (3.6)
U	R: AMP, IPM, TEC, VAN	0	1 (3.6)
W	R: IPM, CIP, LVX, NXN	1 (1.2)	0
X	R: CIP, LVX, NXN, GN-HLR	1 (1.2)	0
Y	R: AMP, TEC, VAN, LIN, KNM	0	1 (3.6)
Z	R: IPM, CIP, LVX, NXN, VAN	0	1 (3.6)
ZX	R: AMP, IPM, CIP, NXN, TEC, VAN	1 (1.2)	0
ZY	R: AMP, IPM, TEC, VAN, GN-HLR, ST-HLR, KNM	1 (1.2)	0

R—resistant, AMP—ampicillin, IPM—imipenem, VAN—vancomycin, GN-HLR—high level resistance to gentamicin, ST-HLR—high level resistance to streptomycin, LIN—linezolid, NFE—nitrofurantoin, LVX—levofloxacin, NXN—norfloxacin, QDF—quinupristin-dalfopristin, KNM—kanamycin, CIP—ciprofloxacin, TEC—teicoplanin

## Assessment of biofilm formation

**Fig. 4:**
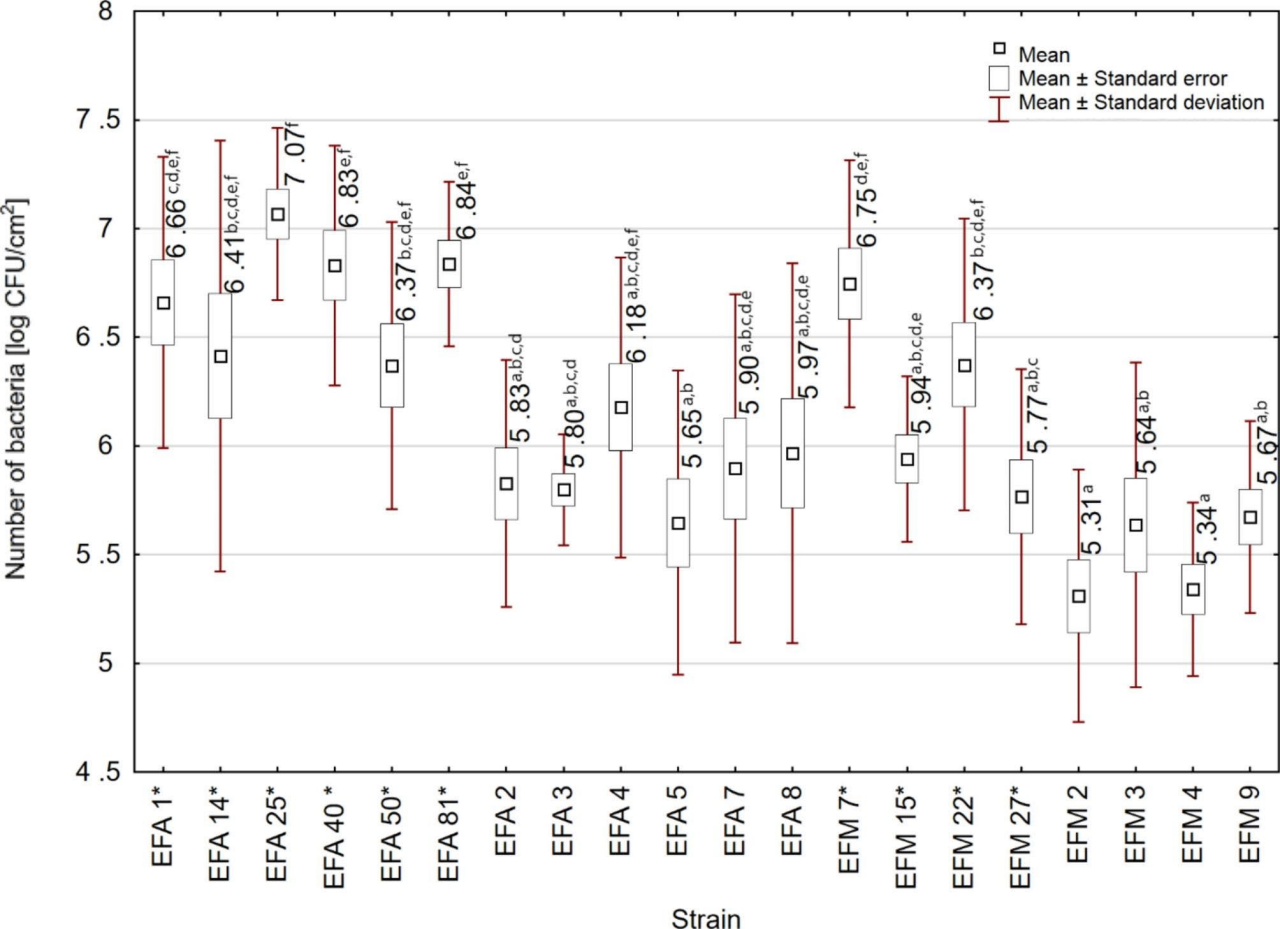
Biofilm formation by *E. faecalis* (EFA) and *E. faecium* (EFM) strains, a,b,c,… –values with different superscript indicate statistically significant differences between strains

The biofilm analysis revealed that all vancomycin-resistant *E. faecalis* and *E. faecium* strains demonstrated a higher biofilm-forming capacity, as compared to the susceptible strains. The lowest cell count of 5.31 log CFU / cm^2^ was reisolated from the biofilm produced by the vancomycin-sensitive strain EFM 2, however it was not statistically different from other susceptible strains of this species. The VRE strain EFA 25 was characterized by the highest capacity for biofilm formation (cell count exceeding 7 log CFU / cm^2^). Its biofilm-forming potential was statistically different from 5 to 6 vancomycin-sensitive *E. faecalis* strains tested. Among the *E. faecium* strains, the vancomycin-resistant strain EFM 7 showed the highest biofilm formation which was significantly different from all vancomycin-sensitive EFM strains (Fig. 4).

## Discussion

Due to the common occurrence of enterococci in the digestive tract of humans and pig, their presence in the pigm farm environment is a naturally occuring phenomenon [23, 24]. Undoubtedly, of particular clinical relevance are two rogue enterococcai species, *E. faecalis* and *E. faecium*. They are commonly isolated from animals, e.g. their fecal specimens and meat as well as from materials and surfaces in animal farms and surrounding environment, means of animal transport, and animal farmworkers [8, 25, 26]. The results of most studies demonstrate that *E. faecalis* is the dominant species in the pig breeding environment. According to Gião et al. [27], of the 249 *Enterococcus* spp. strains isolated from pigs, *E. faecalis* and *E. faecium* accounted for 42.9% and 23.4%, respectively. Tan et al. [14] observed a higher difference in the occurence between *E. faecalis* (73.0%) and *E. faecium* (14.5%) in the material collected from animals, workers and the production environment of seven swine farms in Malaysia. In our study, the occurence frequency of the *E. faecalis* and *E. faecium* was 25.5% and 8.2%, respectively. Contrary results have been reported by de Jong et al. [2018] who isolated a higher number of *E. faecium* strains (328 of the 1146 samples taken from the pig gastrointestinal tract; 28.6%) than *E. faecium* strains (176; 28.6%). The observed differences may depend on many factors, including specific nutritional and hygienic practices implemented in animal farms, climatic conditions, number and source of samples, study area and sampling design. In our study, a much greater number of EFA/EFM-positive samples were fecal specimens, when compared to the study material taken from the production environment. Tan et al. [14] isolated both strains from pig feaces, but identified *E. faecalis* only from samples taken from the pig farm environment. According to studies by Staley et al. [28] *E. faecalis* is more abundant in the intestines of pigs than in faeces, in contrast to *E. faecium*. This was confirmed by Zhao et al. [26] who reported that *E. faecium* isolates accounted for almost 95% of all enterococci found in pig manure. To date, there is not enough research on the diversity of *Enterococcus* spp. strains from pig farms in Poland. Skowron et al. [6] showed that among 195 isolates isolated from pig farms, the largest number belonged to the species *E. hirae* (68%). The other species contributions were: *E. faecalis*—21%, *E. durans*—8% and *E. faecium*—3%. In this study, authors identified 60 genetically distinct strains [6].

The antibiotic resistance of enterococci seems to be a much more serious problem than the very fact of confirming their presence in the environment of farm animals. The global scale of this natural phenomenon is evidenced by the fact that antibiotic-resistant strains have been frequently isolated even from wild animals, theoretically not exposed to contact with chemotherapeutic agents [29, 30]. In the case of antibiotic-resistant zoonotic strains of enterococci, which are opportunistic human pathogens, there is a high risk that they may act as reservoirs of antibiotic resistance encoding genes that could be transmitted to other pathogenic bacteria, including clinically important pathogens [8, 31].

The spectrum of antibiotic resistance of enterococci isolated from the animal breeding environment depends on many factors, including the breeding system, the source of isolates or the specificity of the species and strain [32, 33]. According to the study of Kristich et al. [18], the occurrence of ampicillin resistance is less common in *E. faecalis* than in *E. faecium*. In turn, studies by Aasmäe et al. [34] showed, that the frequency of antibiotic resistant enterococci, including MDR, was higher among isolates obtained from pigs than cattle. Among the *E. faecalis* strains isolated in our study, the highest percentage showed resistance to high-level gentamicin (19.5%). This gentamicin resistance was found only in 17.9% of the *E. faecium* strains. Lee et al. [35] observed resistance to high-level gentamicin in all strains of *E. faecium* and *E. faecalis* derived from finisher pigs. On the contrary, Tan et al. [14] reported that *E. faecali*s strains (68.0%) were more resistant to high-level gentamicin (120 µg), as compared to *E. faecium* (39.0%). In other studies in Poland evaluating the resistance of *Enterococcus* spp. from meat pork, no strains resistant to gentamicin were found [36]. The emergence of high-level gentamicin-resistance (HLGR) in enterococci, first confirmed in *E. faecalis* and then in *E. faecium*, was one of the reasons for the increase in nosocomial infections observed since the 1980s worldwide. Importantly, the HLGR strains have become responsible for a significant proportion of enterococcal bacteremia [37, 38]. The possibility of transferring the HLRG gene from food-derived *E. faecalis* strains to strains colonizing the human gastrointestinal tract was confirmed by Sparo et al. [38].

The occurrence of *E. faecalis* and *E. faecium* strains of the HLSR phenotype among isolated streptomycin-resistant enterococci was similar and amounted to 9.8% and 10.7%, respectively. In the studies of Aasmäe et al. [34], the resistance to streptomycin was found in approximately one-third of porcine enterococci. Jahan et al. [39] showed that streptomycin resistance genes (*aadA*) can be transferred from meat-derived *E. faecium* to clinical *E. faecalis* strains by integron-mediated horizontal gene transfer. Conjugal transfer of HLRS genes (*aadE*) between porcine *E. faecalis* strains was observed by Chotinantakul et al. [40].

From the epidemiological point of view, vancomycin-resistant strains (VRE) constitute the most serious problem related to antibiotic resistance of enterococci [41, 42]. In 2017, vancomycin-resistant enterococci were responsible for approx. 55,000 hospitalizations in the USA, among which over 5,000 cases resulted in death [43, 44]. It has been proven that vancomycin resistance is confirmed less frequently in *E. faecalis* strains than in *E. faecium* isolates [18, 26, 43]. The results of our study are consistent with these observations, where we isolated 6.1% of *E. faecalis* and 14.3% *E. faecium* vancomycin-resistant strains that showed resistance to vancomycin. As reported Lee et al. [35] studying enterococci from finisher pigs, none of the 17 *E. faecalis* strains and only one of the 85 *E. faecium* strains was resistant to this antibiotic. In turn, in the studies reported by Badul et al. [8] and Tan et al. [14], all enterococcal isolates derived from pigs, farm workers as well as different sectors of animal and meat production (animal husbandry, distribution, transport) were vancomycin-resistant.

Regulation and expression of the vancomycin resistance mechanism is related to the presence of specific determinants in the genetic material of enterococci, called *van* operons. Clinically significant isolates are most frequently characterized by the *vanA* and *vanB* genotypes. Recently, despite the persistently higher frequency of *vanA*-positive strains, an increase in the number of *vanB*-positive VRE isolates has been observed [45, 46]. In our study, the genotypic analysis releaved that some *E. faecalis* strains were found to be positive for *vanA* (1; 1.2%), *vanB* (4; 4.9%) and *vanD* (1; 1.2%). For 4 *E. faecium* strains, the presence of the *vanA* (2; 7.1%) and *vanB* (2; 7.1%) genotypes was confirmed. In the studies by Ramos et al. [47], *vanA*-positive strains of *E. feacium* were detected in 18 of the 71 porcine faecal samples (25%), while *vanC* gene was detected in 7 samples (9.9%). The results of the work by Kotzamanidis et al. [48] revealed that 85% of the vancomycin-resistant *E. faecium* isolates from pig feaces were *vanA*-positive. Tan at al. [14] detected *vanB* and *vanC* genes in four enterococcal strains, however the phenotypic testing by disc diffusion method did not confirm their vancomycin resistance. Of a great importance is the fact that in clinical practice *vanA* resistance is induced in the presence of both vancomycin and another glycopeptide antibiotic, teicoplanin. As a consequence, *vanA*-positive strains do not show sensitivity to these both antibiotics, while *vanB*-positive strains are sensitive to teicoplanin [46, 49]. Also in the work of Ramos et al. [47], all *vanA*-positive *E. faecium* strains showed resistance to teicoplanin. In our study, *E. faecalis* and *E. faecium* strains insensitive to teicoplanin accounted for 2.4% and 7.1%, respectively. The results presented by Tan et al. [14] showed the presence of this trait in 2% of the strains of each species. Studies performed in animal farms in South Africa [8] and Australia [35] demonstrated that all *E. faecalis* and *E. faecium* strains isolated from pigs as well as from breeding and meat processing environment were sensitive to teicoplanin.

Resistance to vancomycin, which has undoubtedly contributed to the epidemiological importance of enterococci, is not the only problem raising serious public health concerns. The rapid spread of enterococcal multi-drug resistance forces clinicians to modify the previously established treatment regimens. This phenomenon is found not only in clinical isolates, but also in environmental isolates, including those associated with farm animals. It is believed that one of the reasons for the identification of MDR in enterococci may be an inadequate or irrational therapeutic regimens employed in commercial animal husbandry and breeding [33, 50]. In our study, we distinguished 26 drug susceptibility profiles. The profile A, characterized by sensitivity to all antibiotics and chemotherapeutic agents, was dominant in both *E. faecalis* (67.1%) and *E. faecium* (53.6%) strains, whereas four strains of each species (4.9% and 14.3%, respectively) were multi-drug resistant to 4 or more therapeutics. Gião et al. [27] showed that 27.4% of *E. faecalis* and 4.2% of *E. faecium* isolates from farm animals showed multi-drug resistance to 3 or more antibiotics. However, the results of other studies often report a higher prevalence of MDR strains among enterococci associated with the pig breeding environment. For instance, among all MDR enterococci (resistance to at least three different classes of antibiotics) isolated by Badul et al. [8], multi-drug resistant *E. faecalis* and E. *faecium* accounted for 79.3% (176/222 isolates) and 5.9% (13/222 isolates), respectively. An extremely high, as much as 98%, frequency of occurrence of enterococcal strains insensitive to at least four classes of antibiotics was reported by Tan et al. [14]. The same frequency of multi-drug-resistant enterococcal strains was demonstrated in studies by Lee et al. [35]. More than 94% of *E. faecium* strains were resistant to 3 or more antibiotics, 45.2% of strains showed the resistance to 4 classes of antibiotics, whereas MDR profiles were detected in 76.5% of *E. faecalis* strains. Also in Poland MDR was observed in the 84.6% and 42.5% strains *E. faecium* and *E. faecalis* isolated from pigs, respectively [51]. According to Różańska et al. [36], 56.8% of *E. faecalis* isolates derived from pork meat in Poland were resistant to 3 or more antimicrobials.

In addition, challenges resulting from antibiotic resistance of enterococci may be related to their ability to produce biofilm. The reduced possibility of penetration of antibiotics through the biofilm matrix contributes to the increase in tolerance to antibiotics. Moreover, compared to the planktonic form, biofilm creates more favorable conditions for the horizontal transfer of drug resistance genes [52]. In the studies by Tan et al. [14], biofilm was produced by 62% of enterococcal strains from pigs and humans. Our present study also showed that *E. faecalis* strains exhibit a higher biofilm formation potential than *E. faecium*. Our findings are in accordance with the results obtained by Chotinantakul et al. [40], who reported that at each of the tested temperatures (4 °C, 25 °C, 37 °C), *E. faecalis* produced significantly more biofilm than *E. faecium*. In our study, vancomycin-resistant EFA and EFM strains were characterized by higher potential for biofilm formation compared to non-resistant strains, while Ramadhan and Hegedus [53] did not observe statistically significant differences in biofilm formation beetwen these two strains as well as between vancomycin-resistant and vancomycin-sensitive isolates.

## Conclusions

The irrational use of antibiotics in agriculture and veterinary practice is considered to be one of the key reasons for the rapid spread of antibiotic resistance among microorganisms. The results presented in this work confirm the presence of antibiotic-resistant *E. faecalis* and *E. faecium* strains in pig faeces and various production sectors of the pigsty. Among the isolated *Enterococcus* strains, both vancomycin-resistant and multi-drug resistant strains have been detected. We also found that vancomycin-resistant strains were characterized by a high ability to create biofilms, which facilitates their survival in conditions of increased environmental stress. Owing to the fact that livestock and animal husbandry environment can be a reservoir of antimicrobial resistance and transmission route of antimicrobial resistance genes from commensal zoonotic bacteria to clinical strains, it is of a great importance to public health to monitor trends in this biological phenomenon.

## Materials and methods

### Sample size and sampling procedure

The samples for the study were collected in April 2016 in a piggery in the Kuyavian-Pomeranian Voivodeship. The herd was kept in separate group pens. Each technological group was located in a separate building, and production was carried out in a closed system. The sows were placed in individual pens 7 days before farrowing. The average cast comprised of 567 sows and gilts, 2303 piglets, 1719 weaners and porkers, and 15 boars. The material consisted of samples in the form of swabs from troughs, feeding passages and corridors, manure channels, and faeces samples collected from various production sectors of the pigsty: the suckling pigs sector, weaned pigs sector, piglets and porkers sectors, and mating area. Additionally, samples were taken from the loading ramp and platform. In total, 475 samples were collected from the animal husbandry environment in the form of swabs (n = 350) and weights of faeces (n = 125). The swabs were collected using a sterile gauze (5 cm × 5 cm) held by tweezers. In order to standardize the sampling procedure, sterile cardboard templates with an area of 10 × 10 cm were used. Swabs from the troughs, feeding passages, coorridors and manure channels were collected from 5 separate places along the axis of the buildings, and swabs from the pens were collected from the center and each corner. Only pens with healthy animals were included in the study. Faecal samples (10 g) were collected from fresh fecal material.

### Isolation of ***Enterococcus*** spp. strains

In the first stage of the laboratory tests, the samples were pre-diluted. 10 g of faeces or swab fragments were placed in 25 mL Ringer’s solution (potassium chloride: 0.30 g/L, calcium chloride dihydrate: 0.33 g/L, sodium chloride: 8.60 g/L) and shaken for 30 min. Then 10 mL of the resulting suspension was transferred to 90 mL of azide dextrose broth (Merck). The inoculated media were incubated at 37 °C for 48 h. After that, the inoculum was streaked on kanamycin esculin azide agar (Merck) and incubated at the same conditions. Enterococci grew in the form of tiny gray colonies surrounded by an olive-black agar zone. Single colonies from the resulting cultures were plated on Standard I Nutrient Agar (Merck) and incubated at 37 °C for 24 h. The resulting bacterial colonies were then suspended in nutrient broth (Merck) supplemented with 15% glycerol (Avantor) and stored at -80 °C until use.

### Identification of ***Enterococcus*** species

For species identification, the frozen strains were plated on nutrient agar (Merck) and incubated for 48 h at 37 °C. After that, the strains were plated again onto nutrient agar (Merck) and incubated under the same conditions. DNA was isolated from the grown colonies by a spin column method using the Genomic Mini AX Bacteria Spin Kit (A&A Biotechnology), according to the manufacturer’s instructions. The isolated DNA served as template in the PCR reaction performed with the use of primers specific to 16 S rRNA gene (for identification of the genus *Enterococcus* spp.) and primers specific to *sodA* gene encoding superoxide dismutase (for strain classification). The following reference strains were used in the study: *Enterococus faecium* PCM 1859 and *Enterococcus faecalis* PCM 2673. The PCR reaction was performed according to the procedure described previously [54] (Table 4).

**Table 4:** Characteristics of primers used in the *Enterococcus* species identification [49]

Species	Primer name	Primer sequence	Product size [bp]
*Enterococcus* spp.	16S1 16S2	5’-AACGCGAAGAACCTTAC-3’ 5’-CGGTGTGTACAAGACCC-3’	440
*E. faecalis*	FL1 FL2	5’-ACTTATGTGACTAACTTAACC-3’ 5’-TAATGGTGAATCTTGGTTTGG-3’	360
*E. faecium*	FM1 FM2	5’-GAAAAAACAATAGAAGAATTAT-3’ 5’-TGCTTTTTTGAATTCTTCTTTA-3’	215

The composition of the reaction MasterMix was as follows: 1×DreamTaq polymerase buffer, 16 µM of each primer, dNTPs mix (0.2 mM each), 3.5 U of DreamTaq polymerase, 3.0 mM MgCl_2_ (all reagents were purchased from Fermentas). Next, 3.0 µL of the isolated DNA were added. The total volume of the reaction mixture was 25 µL. The amplification conditions were as follows: initial denaturation step at 95 °C for 4 min, followed by 30 cycles of denaturation at 95 °C for 30 s, annealing at 55 °C for 60 s and elongation at 72 °C for 60 s. The final extension step was performed at 72 °C for 7 min.

Electrophoretic separation of the PCR products (7 µL) was performed in a 2% agarose gel in 1×TBE buffer (EURx). In order to visualize the DNA fragments, a non-specific DNA intercalating dye SimpleSafe (EURx) was used. In order to assess the size of the PCR products, the GeneRuler 100 bp DNA Ladder (ThermoScientific) with a range of 100 to 1000 bp was used as a DNA molecular weight marker. The DNA electrophoresis was carried out at 20 V for 10 min, and then at 85 V for 110 min. The gel were visualized and documented using a UV transilluminator (BioRad).

### Analysis of genetic similarity

In order to determine the degree of genetic similarity of the tested *Enterococcus* strains, the Random Amplified Polymorphic DNA (RAPD) method was employed with the use of a nine deoxyribonucleotide primer of the following sequence: 5’-ACGCGCCCT-3’ [55]. The composition of the reaction MasterMix was as follows: 1×DreamTaq polymerase buffer containing 1.5 mM MgCl_2_, 5 µM primer, dNTPs mix (0.2 mM each), 2.5 U of DreamTaq polymerase (all reagents were purchased from Fermentas). 22 µL of MasterMix per sample was pipetted into sterile 200 µL PCR tube strips, followed by addition of 3.0 µL of the isolated DNA. The total volume of the reaction mixture was 25 µL. The amplification conditions were as follows: 4 cycles of denaturation at 94 °C for 45 s, annealing at 30 °C for 120 s and elongation at 72 °C for 60 s, followed by 26 cycles of denaturation at 94 °C for 5 s, annealing at 36 °C for 30 s and elongation at 72 °C for 30 s. The final extension step was performed at 72 °C for 10 min.

Electrophoretic separation of the PCR products (8 µL) was performed in a 1.5% agarose gel in 1×TBE buffer (EURx). The GeneRuler 100 bp DNA Ladder (ThermoScientific) with a range of 100 to 1000 bp was used as DNA molecular weight marker. DNA electrophoresis was carried out at 20 V for 10 min, and then at 85 V for 90 min. DNA visualization and gel documentation were carried out as described above.

Data matrices were prepared for individual isolates in order to document the location of the RAPD reaction products. Then, the degree of genetic relationship between isolates of a given species was determined. For this purpose, phylogenetic trees were plotted in the STATISTICA 11 PL (StatSoft) program, using the UPGMA clustering method and Dice’s similarity coefficient as a distance unit.

### Analysis of antibiotic resistance

The antibiotic susceptibility of the identified *Enterococcus* spp. strains was determined using the disc diffusion method. Following the microbial culture for 24 h at 37 °C, isolate suspensions were prepared in 0.9% saline, with the standard density of 0.5 McFarland, and subsequently spread on Mueller-Hinton agar (MHA, bioMérieux). Antimicrobial discs were applied and pressed firmy onto the agar surface with sterile forceps. The sensitivity to the following antibiotics was assessed: ampicillin (2 µg), imipenem (10 µg), ciprofloxacin (5 µg), levofloxacin (5 µg), norfloxacin (10 µg), teicoplanin (30 µg), vancomycin (5 µg), and quinupristin-dalfopristin (15 µg) for *E. faecium*; and tigecycline (15 µg), linezolid (10 µg), and nitrofurantoin (100 µg) for *E. faecalis*. Additionally, in order to detect HLGR (high-level gentamicin-resistant) and HLSR (high-level streptomycin-resistance) phenotypes, discs with gentamicin (30 µg) and streptomycin (300 µg) were applied. The dishes were incubated at 35 °C for 20 h. The results were interpreted in accordance with the EUCAST v. 7.0 recommendations [56].

In addition, minimal inhibitory concentrations (MICs) have been determined for vancomycin, teicoplanin, gentamicin, streptomycin and kanamycin. The MIC determination was carried out for the tested *Enterococcus* spp. strains using the agar microdilution method in a 96-well titration plate format, according to the CLSI (Clinical and Laboratory Standards Institute) recommendations. Strains were incubated in MHA at 37˚C for 24 h, then cultured in Tryptic Soy Broth (TSB, BioRad) under the same conditions. Then the bacteria were centrifuged at 4000 rpm for 15 min, the supernatant was removed by decantation, and the cell pellet was suspended in 4 mL of MHB at a 0.5 McFarland density. Inoculum suspensions were further diluted 1:100 in Mueller-Hinton broth before inoculation and 100 µl of the suspension were placed on the plate in triplicate. A two-fold serial dilution of tested antibiotics ranging from 0.25 to 2048 µg/mL was prepared in 100 µL of sterile MHB medium. The titration plates were placed in a humidity chamber and incubated at 37˚C for 24 h. Aftewards, suspension turbidity was measured according to the EUCAST guidelines [56]. *Enterococcus faecalis* ATCC 29,212 was used for quality-control purposes. The obtained results were used to determine the phenotypes of glycopeptide resistance, HLGR, HLSR and kanamycin resistance. Determination of the Van phenotype for *Enterococcus* spp. strains is based on the determination of the MIC values for vancomycin and teicoplanin. The literature specifies the concentration ranges of both antibiotics within which the established MIC values must fall. On this basis, the Van phenotype is assigned. In turn, determining the genotype consists of detecting the appropriate *van* gene [20]. The results were interpreted on the basis of the EUCAST recommendations [56] and literature [20] summarized in Table 5.

**Table 5:** Characteristics of glycopeptide resistance phenotypes for the genus *Enterococcus* [20]

Antibiotic	Resistance phenotype				
	VanA	VanB	VanD	VanE	VanG
	MIC (mg/L)				
Vancomycin	60–1000	4–1000	64–128	8–32	≤ 16
Teicoplanin	16–512	0.5–1	4–64	0.5	≤ 0.5

### Detection of glycopeptide resistance genotypes

In order to determine the glycopeptide resistance genotypes in the tested *Enterococcus* spp. strains, the multiplex PCR reaction was performed with the use of isolated DNA (previously described in the section about identification of *Enterococcus* species). Details of primers used in the analysis are listed in Table 6.

**Table 6:** Characteristics of primers used in the identification of glycopeptide resistance genotypes

Genotype	Primer name	Primer Sequence	Product size [bp]	References
*vanA*	EA-F	5’-GGGAAAACGACAATTGC-3’	732	[57]
	EA-R	5’-GTACAATGCGGCCGTTA-3’		
*vanB*	EB-F	5’-ACGGAATGGGAAGCCGA-3’	647	[58]
	EB-R	5’-TGCACCCGATTTCGTTC-3’		
*vanD*	ED-F	5’-TGTGGGATGCGATATTCAA-3’	500	
	ED-R	5’-TGCAGCCAAGTATCCGGTAA-3’		
*vanE*	EE-F	5’-TGTGGTATCGGAGCTGCAG-3’	430	
	EE-R	5’-ATAGTTTAGCTGGTAAC-3’		
*vanG*	EG-F	5’-CGGCATCCGCTGTTTTTGA-3’	941	
	EG-R	5’-GAACGATAGACCAATGCCTT-3’		

The reference strains of *E. faecium* ATCC 700,221 (*vanA*) and *E. faecalis* ATCC 51,299 (*vanB*) were used in the study. Due to the lack of access to reference strains of other VRE genotypes, the control strains were selected based on the phenotype. The composition of PCR reaction mixture and amplification conditions were established according to the work by Depardieu et al. [58]. The composition of the reaction MasterMix was as follows: 1×DreamTaq polymerase buffer, 0.5 µM of each primer, dNTPs mix (0.2 mM each), 2.0 U of DreamTaq polymerase, and 3.0 mM MgCl_2_ (all reagents were purchased from Fermentas ).The 3.0 µL of the isolated DNA were added. The total volume of the reaction mixture was 25 µL. The amplification conditions were as follows: initial denaturation step at 95 °C for 3 min, followed by 30 cycles of denaturation at 94 °C for 60 s, annealing at 54 °C for 60 s and elongation at 72 °C for 60 s. The final extension step was performer at 72 °C for 7 min. Electrophoretic separation of the PCR products was performed as described above (see Identification of *Enterococcus* species).

### Assessment of biofilm formation on a stainless-steel surface

The assesment of biofilm formation was performed in accordance with previous studies [59]. Sterile steel coupons (1 cm × 2 cm), three replications for each strain, were placed into tubes containing 3 mL of bacterial suspension in BHI (Merck) with standard density of 0.5 McFarland (1.5 × 10^8^/mL) and incubated aerobically at 37 °C for 72 h. Six *E. faecalis* (EFA 1, 14, 25, 40, 50, 81) vancomycin-resistant strains (VRE), four *E. faecium* (EFM 7, 15, 22, 27) VRE strains, six vancomycin-susceptible EFA (2, 3, 4, 5, 7, 8) and four EFM (2, 3, 4, 9) strains were used for biofilm formation studies. Strains were randomly selected from each group. The medium was changed every 24 h. At each medium change, the coupons were rinsed with phosphate buffered saline (PBS) (137 mM NaCl, 2.7 mM KCl, 8 mM Na_2_HPO_4_, 2 mM KH_2_PO_4_) (BTL). After incubation, the samples were rinsed with PBS and placed into a new tube containing 3 mL of PBS. Next, sonication was performed using the Ultrasonic DU-4 sonicator (Nickel-Electro Ltd.) (10 min, 30 kHz, 150 W). After that, serial 10-fold dilutions of the obtained suspension were prepared, plated on the Columbia Agar medium with 5% Sheep Blood (Becton Dickinson) and incubated at 37 °C for 24 h. As positive control, a strong biofilm-forming strain *Staphylococcus aureus* ATCC 35,556 was used [60, 61]. Stainless steel fragments incubated in the sterile BHI medium only served as negative control. The results were expressed as the log CFU × cm^2^.

### Statistical analysis

The data are expressed as mean ± standard error of mean (SEM) or standard deviation (SD). The results were obtained from at least three technical replicates. To determine the statistical significance, analysis of variance (ANOVA) followed by the Tukey post hoc test for multiple comparisons was performed. Statistical analysis and data visualization were performed with the Statistica 12 PL (StatSoft) software. A p-value < 0.05 was considered statistically significant.

## Data Availability

The datasets used and/or analysed during the current study available from the corresponding author on reasonable request.
